# Adipose-derived regenerative cell (ADRC)-enriched fat grafting: optimal cell concentration and effects on grafted fat characteristics

**DOI:** 10.1186/1479-5876-11-254

**Published:** 2013-10-10

**Authors:** Natsuko Kakudo, Yoshihito Tanaka, Naoki Morimoto, Takeshi Ogawa, Satoshi Kushida, Tomoya Hara, Kenji Kusumoto

**Affiliations:** 1 Department of Plastic and Reconstructive Surgery, Kansai Medical University, Osaka, Japan

## Abstract

**Background:**

To overcome the absorption of traditional fat grafting, techniques for adipose-derived regenerative cell (ADRC)-enriched fat grafting are currently being adapted for practical application. The Celution®800/CRS (Cytori Therapeutics, San Diego, CA) has enabled rapid grafting of the patient’s own freshly harvested ADRCs without requiring a culturing step. However, the optimal cell concentration and the effects of ADRCs on the characteristics of grafted fat after free fat grafting remain unclear.

**Methods:**

ADRCs were isolated and purified from human fat tissue using the Celution®800/CRS. Animals that received fat grafting without the addition of ADRCs were designated the control group (group A). The number of ADRCs per grafted fat volume (mL) was adjusted to 3 × 10^5^, 1.5 × 10^6^, and 3 × 10^6^ cells/mL (groups B, C, and D, respectively), mixed with free fat, and transplanted as ADRC-enriched fat grafting. These mixtures were transplanted subcutaneously into BALB/C Jcl-nu/nu mice. The volume of grafted fat was determined 5 months after transplantation, and histological assessments were performed.

**Results:**

ADRC-enriched fat grafting resulted in decreased fat absorption and the formation of greater numbers of new blood vessels in the grafted fat. The optimal ADRC concentration in this study was found to be 3 × 10^5^ cells/mL (group B), with higher concentrations resulting in increased cyst and fibril formation in the grafted fat.

**Conclusions:**

This study used the Celution®800/CRS for free fat grafting and demonstrated that the concentration of transplanted ADRCs affected the engraftment and quality of the grafted fat.

## Introduction

Autologous free fat grafting is a widely accepted technique used for the correction of various soft-tissue defects because it is biocompatible, versatile, natural-looking, non-immunogenic, inexpensive, and readily obtainable with low donor site morbidity [1,2]. Although free fat grafting is used as a method for soft tissue reconstruction, absorption of the fat after grafting is problematic. Free fat grafting often has a low survival rate, and adipose tissue can be quickly resorbed and replaced by fibrous tissue and oil cysts [3,4]. To overcome this shortcoming of traditional fat grafting, techniques for adipose-derived regenerative cell (ADRC)-enriched fat grafting are currently being adapted for practical application. ADRCs are a heterogeneous or mixed population of cells found in adipose tissue by collagenase digestion [5]. This population includes adult stem cells, endothelial progenitor cells, endothelial cells, vascular smooth muscle cells, and leukocytes [5]. Recent studies that have investigated fat grafting enriched with cultured ADRCs have indicated that the technique may be valid and reproducible, and it may result in increased graft viability as assessed by improved transplant volume and histology [6,7].

The Celution®800/CRS (Cytori Therapeutics, San Diego, CA) was recently developed to enable rapid grafting of the patient’s own freshly harvested ADRCs by automating and standardizing the extraction, washing, and concentration of ADRCs for clinical use without requiring a culturing step [5]. The risk of infection and degeneration was shown to be very low because no culture was necessary, and establishing a new cell processing center (CPC) was also unnecessary. Accordingly, no cost for maintenance or labor is necessary, which makes it superior for cost performance, and a delivery platform for regenerative medicine can be prepared at a low cost to perform cell therapy. Lin et al. reported the Celution output from 6 patients as 2.95 × 10^5^ ADRCs per mL of lipoaspirate, with a mean viability of 86.6% [5]. Furthermore, clinical trials of free fat grafting using this device were performed in Europe [8]. The Celution®800/CRS for free fat grafting is gaining recognition because the system provides clinical researchers with a ‘real-time’ treatment setting that is cost-effective and safe. However, the optimal cell concentration and the effects of ADRCs on the characteristics of grafted fat after free fat grafting remain unclear.

The purpose of this study was to investigate the optimal cell concentration of human ADRCs isolated by the Celution®800/CRS and their effects on the characteristics of grafted fat after free fat grafting in a nude mouse fat transplantation model.

## Materials and methods

### The celution system and ADRC isolation

**Figure 1:**
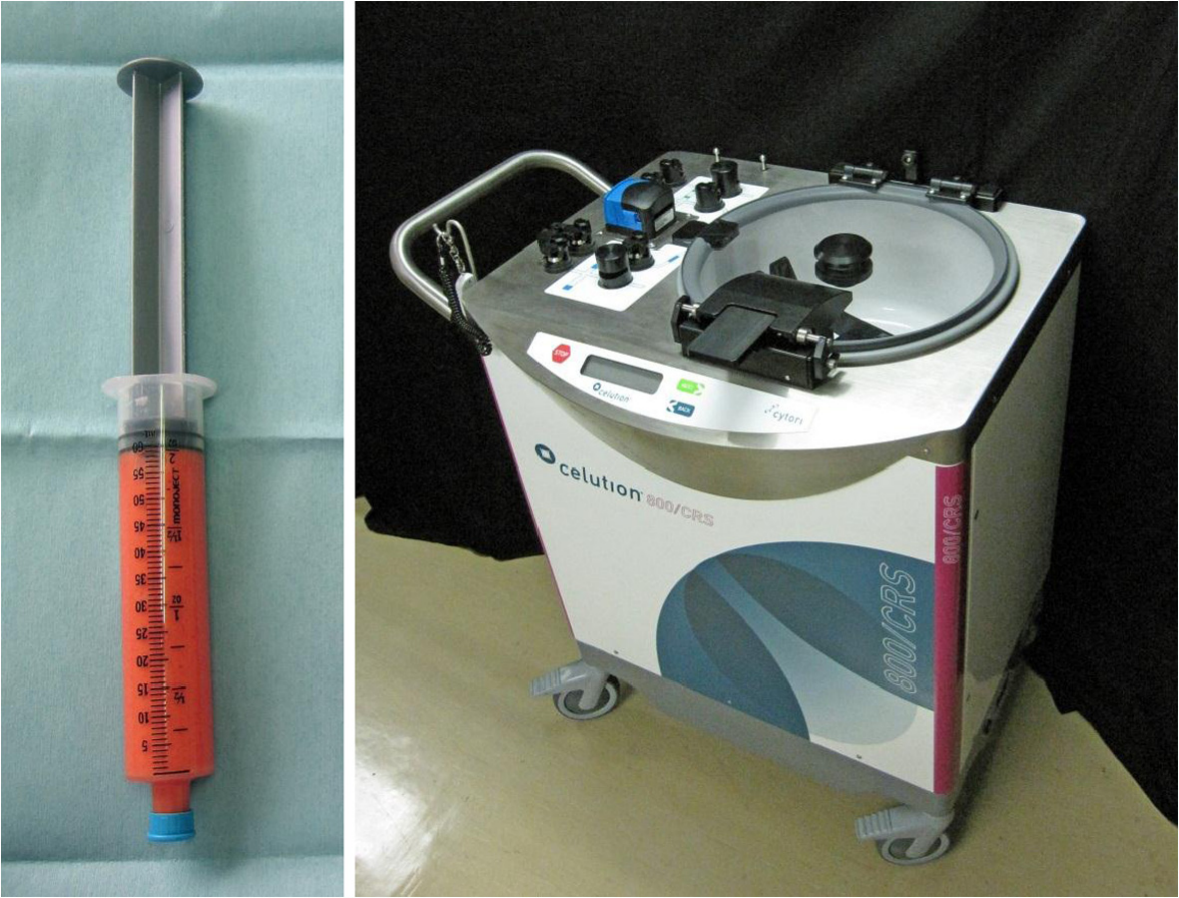
**Human abdominal subcutaneous fat (left) was applied to the Celution**®**800/CRS (right) to extract ADRCs.**

The Celution®800/CRS system was used in this study to extract ADRCs from human abdominal subcutaneous fat tissue (Figure 1). The system consisted of two parts: one for tissue washing and digestion, and the other for cell concentration. Basically, the whole system automates the manual isolation steps modified from Zuk et al. [7]. Adipose tissue is introduced into the device, subsequently washed to remove red blood cells and debris, and then enzymatically digested. After adipose tissue digestion and release of mononuclear cells from the adipose tissue matrix, the released cells are transferred into the centrifuge processing part automatically. The suspension cells are concentrated by centrifugation and wash cycles. These cycles were repeated until the entire volume of the input cell suspension had been processed and the desired cell population had been localized into the output chamber and reduced to a minimal volume [5]. The cells were washed one final time by lactated Ringer’s solution, and they were then ready for use. The whole system can be operated aseptically with the use of clinical-grade solutions, such as saline and lactated Ringer’s, and single-use Celution™ consumable sets.

Human abdominal subcutaneous fat (female, 50s) was collected from excess tissues excised by plastic and reconstructive surgery. The subject provided informed consent, and an Institutional Review Board-approved protocol was used. Cell isolation was performed by the programmed sequence set in Celution®800/CRS according to the manufacturer’s protocol. The digestion enzyme used in this study was Celase™ (Cytori Therapeutics). The washed digestion solution used was a lactated Ringer’s solution called Lactec G injection (Otsuka Pharmaceutical Factory Inc., Naruto, Japan). The disposable kit, including the tissue wash and digestion containers and cell concentration chambers, were sterile and accompanied the Celution®800/CRS.

### Preparation of ADRC-enriched fat grafting

**Figure 2:**
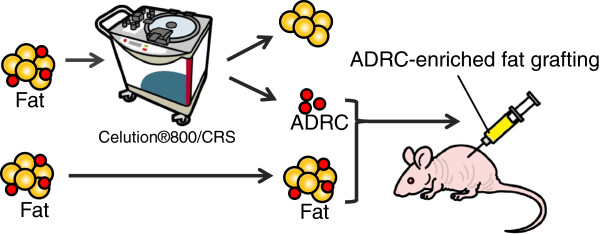
**Experimental protocol.** ADRCs extracted using the Celution®800/CRS were mixed with fat and subcutaneously implanted in the dorsal region of nude mice for ADRC-enriched fat grafting.

**Figure 3:**
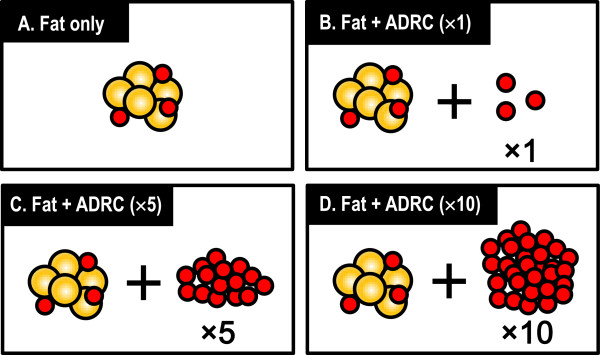
**Animals that received free fat grafting without the addition of ADRCs were designated the control group (group A).** The number of ADRCs per grafted fat volume (mL) was adjusted to 3 × 10^5^, 1.5 × 10^6^, and 3 × 10^6^ cells/mL (groups **B**, **C**, and **D**, respectively), mixed with 0.3 mL of free fat, and transplanted for ADRC-enriched fat grafting. ADRC-enriched fat grafts for groups **B**, **C**, and **D** contained 2, 5, and 10 times more ADRCs than those in grafts in group **A**, respectively.

The ADRC-enriched fat grafting procedure is explained in Figure 2. Human fat tissue was divided into two parts, with one used for extraction and the other used for fat grafting. The cell viability and concentration of ADRCs from the Celution®800/CRS were calculated by trypan blue staining using a hemocytometer, with dead cells stained blue and live cells unstained. The number of cells and viability were measured 5 times. The animals allocated for 0.3 mL of fat grafting without the addition of ADRCs were designated the control group (group A). The ADRC numbers were adjusted to 3 × 10^5^, 1.5 × 10^6^, and 3 × 10^6^ cells/mL (groups B, C, and D, respectively) mixed with 0.3 mL of free fat, and transplanted for ADRC-enriched fat grafting. A preliminary experiment confirmed that 3 × 10^5^ ADRCs could be extracted from 1 mL of fat. Therefore, ADRC-enriched fat grafts containing 1, 5, and 10 times more ADRCs than those in the grafts in group A were prepared for groups B, C, and D, respectively (Figure 3).

### Animal model

All animal procedures were performed in accordance with the guidelines of the Kansai Medical University Animal Care and Use Committee. Animals were caged individually, and their environment was maintained at room temperature with a 12-h day/night (light/dark) cycle. Standard laboratory food for rats and water were freely provided to the animals. The Coleman technique [9] was used for free fat grafting in this study. Therefore, 6-week-old BALB/cAJcl-nu/nu mice (n=6) were anesthetized with isoflurane, and each mouse was injected on the back subcutaneously at one spot with ADRC-enriched fat using a 14-G needle. The transplants were excised and analyzed after 5 months as described below.

### Volume measurement of transplanted fat tissue

Transplanted fat tissues were excised carefully, and volumes were measured using the liquid overflow method [10], as advocated by Ayhan et al. for animal free fat grafting.

### Observations using light microscopy

Fat grafts in each mouse were fixed in 10% formalin neutral buffer solution (pH 7.4), and demineralized with EDTA for 24 h. The sections of the implants, 4 μm in thickness, were mounted on glass slides and rehydrated. Sections were prepared for hematoxylin and eosin (HE) staining, Azan Mallory staining, and immunostaining.

The histological parameters evaluated were as follows, according to the method previously described by Shoshani et al. [11]: the presence of intact and nucleated fat cells; the presence of cysts and vacuoles; inflammation, as evidenced by the infiltration of lymphocytes and macrophages; and the presence of fibrosis and other components of connective tissue (i.e., collagen and elastic fibrils). Briefly, each of these parameters was graded on a semiquantitative scale of 0 to 5 by evaluating the relative presence of each of the histological parameters under examination as follows: 0 = absence; 1 = minimal presence; 2 = minimal to moderate presence; 3 = moderate presence; 4 = moderate to extensive presence; and 5 = extensive presence. HE-stained preparations were used to evaluate cysts/vacuoles and inflammation, and Azan Mallory-stained preparations were used to evaluate fibrosis. All evaluations were carried out blindly by two of the authors (Kakudo and Kushida).

### Immunolocalization of von willebrand factor

To study whether ADRCs could promote neovascularization in the fat grafts, the capillary areas in the grafts were identified using immunostaining for von Willebrand factor, an endothelial cell marker. Deparaffinized sections were used for immunostaining of von Willebrand factor. Before the primary antibody was added, antigenic sites were treated with proteinase K. The slides were immersed in 3% hydrogen peroxide solution for 10 min to inhibit endogenous peroxidase before incubating for 5 min in Tris-buffered saline T (TBST) (50 mM Tris–HCl, pH 7.6, 0.15 M NaCl + 0.05% Tween).

To detect von Willebrand factor immunoexpression, slides were then exposed to rabbit polyclonal anti-human von Willebrand factor antibody (DakoCytomation, Inc., Carpinteria, CA), diluted 1:5000 in phosphate-buffered saline (PBS), at 4°C overnight. After washing in TBST, EnVision™+ HRP, Rabbit (Dako North America, Inc., Carpinteria, CA) was added to the slides for 30 min at room temperature. After washing in TBST, the slides were exposed to the Liquid DAB+Substrate Chromogen System (DakoCytomation, Inc.) according to the manufacturer’s protocol. Sections were then counterstained with Mayer hematoxylin, cleared, and mounted with a cover slip. Histological evaluations of 10 fields per section taken from the center of surviving fat tissue were used to determine the area of the capillaries (μm^2^/field), which is an index of neovascularization.

### Statistical analysis

The Mann–Whitney U test was used for comparisons between groups, with p < 0.05 being regarded as significant. Data are presented as means ± S.D.

## Results

### Cell viability and cell concentration of ADRCs from the celution system

ADRC viability was 93% ± 2%. Using the Celution®800/CRS, 3.0 × 10^5^ ± 5.4 × 10^4^ ADRCs were extracted from 1 mL of fat.

### Assessment of fat tissue survival

**Figure 4:**
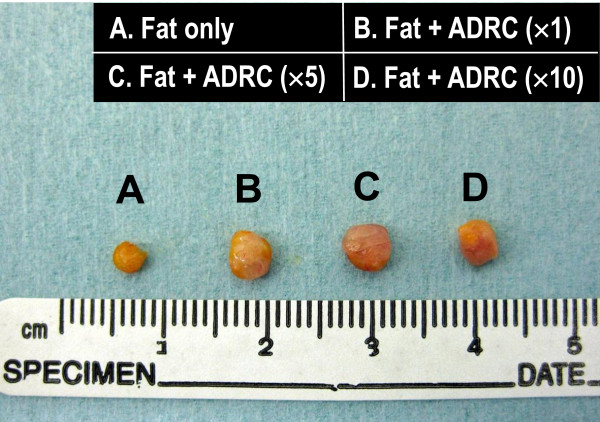
**Surviving transplanted fat preparations under the skin of representative BALB/c Jcl-nu/nu mice.** Regarding the group that received fat grafting without the addition of ADRCs as a control group (group **A**), 3 × 10^5^, 1.5 × 10^6^, and 3 × 10^6^ ADRCs per transplanted fat volume (mL) were added to groups **B**, **C**, and **D**, respectively.

**Figure 5:**
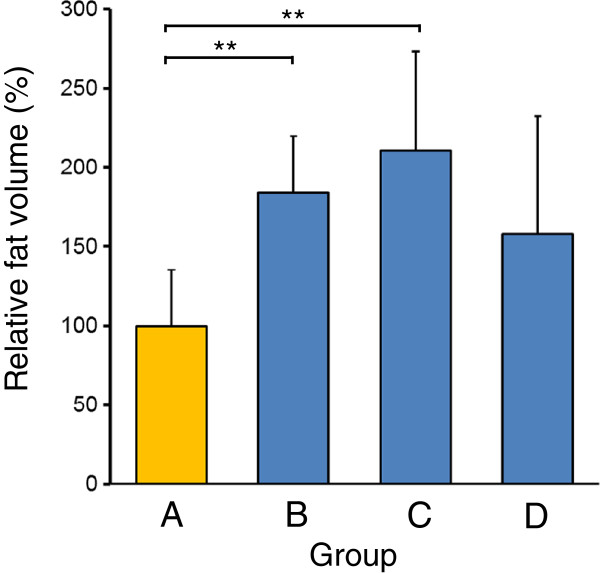
**Transplant volumes were measured by the liquid overflow method**, **and this was used to calculate the relative fat volume (%) compared with group A.** Fat absorption was significantly less in groups **B** and **C** than in group **A**.

Animals that received fat grafting without the addition of ADRCs were designated the control group (group A). The number of ADRCs per grafted fat volume (mL) was adjusted to 3 × 10^5^, 1.5 × 10^6^, and 3 × 10^6^ cells/mL (groups B, C, and D, respectively), mixed with free fat, and subcutaneously transplanted. None of the animals died over the 5 months after transplantation. Transplanted fat was extirpated at 5 months, as shown in Figure 3. Transplant volumes were measured by the liquid overflow method, and this was used to calculate the relative fat volume (%) compared with group A (Figure 4). The relative fat volumes for groups A through D were 100.1% ± 35.7%, 184.1% ± 35.6%, 210.4% ± 62.1%, and 157.8% ± 72.2%, respectively. Relative fat volumes were significantly higher in groups B and C than in group A, which showed that fat absorption was inhibited (Figure 5).

### Histological evaluation of fat transplants

**Figure 6:**
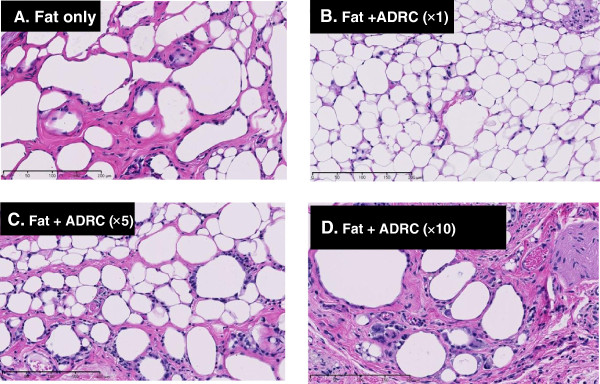
**Histological evaluation of transplanted fat tissue 5 months after transplantation using hematoxylin-eosin staining Bar, 200 μm.** Equivalent cysts and inflammatory reactions were noted in groups **A** and **C**. In group **B**, the transplanted fat tissue was mostly occupied by normal fat. Cysts, inflammatory reactions, and fibrosis were noted at a high rate in group **D**.

**Figure 7:**
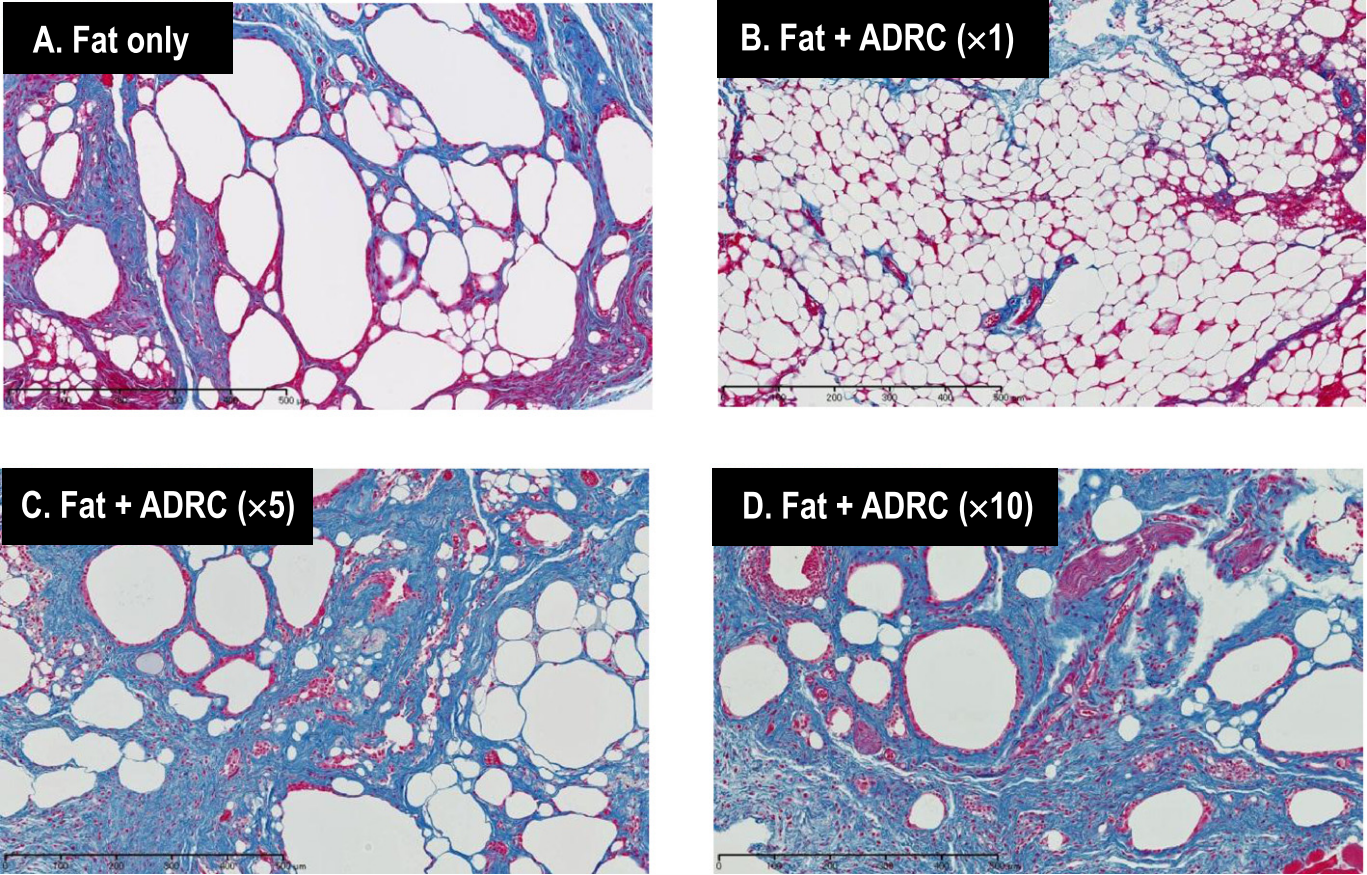
**Histological evaluation of transplanted fat tissue 5 months after transplantation using Azan Mallory staining Bar, 500 μm.** An equivalent amount of fibrosis is observed in groups **A** and **B**. A larger amount of fibrosis is noted in groups **C** and **D** than in group **A**.

Cysts/vacuoles and inflammation were evaluated by HE staining, and collagen fibers were evaluated by Azan Mallory staining. Cyst formation between normal fat was noted in group A, and inflammatory cells were present around the cysts. In group B, the majority of adipocytes was similar to normal adipocytes. Cysts and inflammatory cells were present in group C, and fibrosis was noted between the adipocytes. In group D, many inflammatory cells and fibrosis were present between large cysts (Figures 6 and 7). Cysts/vacuoles and fibrosis were more common in Groups C and D than in Group B. The fat cells in Group B were uniformly sized and finely structured, similar to those in normal fat tissue.

**Figure 8:**
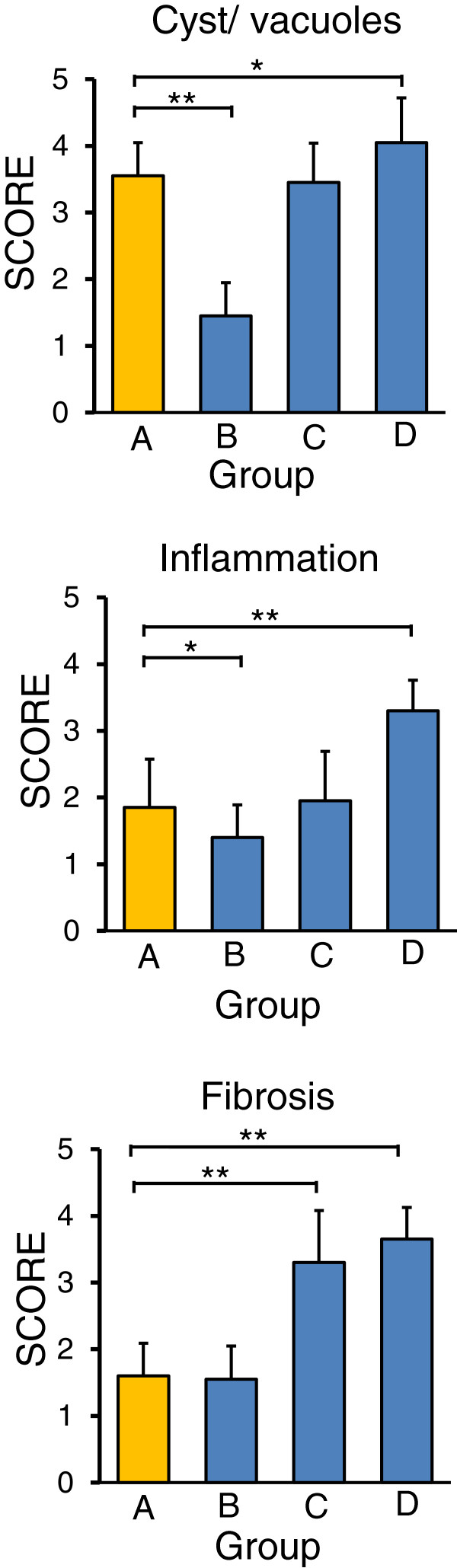
**The number of cysts/vacuoles is significantly lower in group B than in group A, but is higher in group D (upper).** Inflammation is significantly inhibited in group **B** relative to group **A**, but is enhanced in group **D** (middle). The amount of fibrosis in group **B** is similar to that in group **A**, but is significantly higher in groups **C** and **D** (lower).

The results of the histologic evaluation score were consistent with the above findings. The number of cysts/vacuoles was significantly lower in group B than in group A, but higher in group D. Inflammation was significantly inhibited in group B relative to group A, but was enhanced in group D. The amount of fibrosis in group B was similar to that in group A, but was significantly higher in groups C and D (Figure 8).

### Evaluation of vascularization

**Figure 9:**
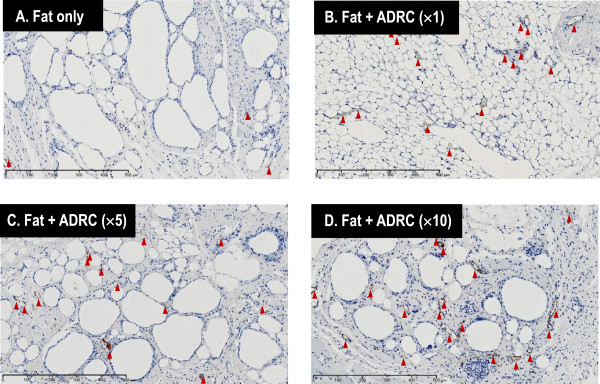
**Vascularization was evaluated by immunostaining of von Willebrand factor.** The amount of new blood vessels formed is higher in groups **B**, **C**, and **D** than in group **A** (Bar, 500 μm).

**Figure 10:**
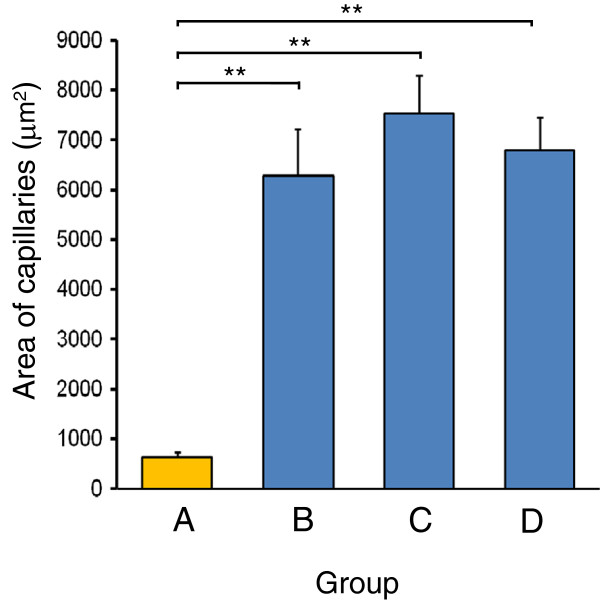
**Area of capillaries in surviving transplanted fat tissue.** Histological evaluations of 10 fields per section taken from the center of the surviving fat tissue show that the capillary area is higher with ADRC-enriched fat grafting (groups **B**, **C**, and **D**) than with fat only (group **A**).

In group A, a few blood vessels were present in the interstitial tissue between adipocytes. Many new blood vessels formed between normal adipocytes in group B. Many blood vessels were noted around cysts and between fibrous tissues in groups C and D (Figure 9). Vascularization was significantly higher in groups B, C, and D than in group A (Figure 10).

## Discussion

This study demonstrated that fresh ADRC-enriched fat grafting using the Celution®800/CRS results in decreased fat absorption and formation of greater numbers of new blood vessels in the grafted fat. The optimal ADRC concentration in this study was found to be 3 × 10^5^ cells/mL, with higher concentrations resulting in increased cyst and fibril formation in the grafted fat. We concluded that the concentration of transplanted ADRCs affects the engraftment and quality of the grafted fat.

Autologous fat grafting for the correction of soft-tissue defects is being increasingly used in plastic and reconstructive surgery. The main disadvantage of this technique is the high absorption rate of the transplanted fat, which can reach 70% of the volume; therefore, overcorrection and repeated operations are needed [12]–[14]. It has been reported that fat grafting can be quickly resorbed and in part replaced by fibrous tissue and oil cysts [3,12]. To overcome these disadvantages, several studies have developed new ways to increase the viability of transplanted fat tissue. It has been reported that ADRCs contain several types of ADRCs and regenerative cells, which may help improve graft retention through multiple mechanisms [7]. For example, Matsumoto et al. reported that, when aspirated fat was transplanted subcutaneously into severe combined immunodeficiency mice with (cell-assisted lipotransfer; CAL) or without (non-CAL) vascular stromal fractions containing human ADRCs isolated from adipose tissue, the CAL fat survived better (35% larger on average) than the non-CAL fat, and microvasculature was detected more prominently in the CAL fat, especially in the outer layers [13]. Although the present study supported their results, they had extracted ADRCs manually, whereas an entirely automated extraction device was used in the present study. Moseley et al. also reported that when fat removed by lipectomy from Rosa mice with or without freshly isolated, non-cultured ADRCs was transplanted in the subdermal scalp space of SCID mice, the mass of fat was significantly greater with freshly isolated ADRCs than with adipose tissue alone [14]. Recently, Zhu et al. demonstrated that, when inguinal fat pads from B6129SF1/J mice with or without ADRCs from Rosa mice were implanted in the subdermal scalp space, the mass of fat enriched with ADRCs was about two-fold higher than control at both 6 and 9 months of follow-up [7]. Although the usefulness of ADRCs in inhibiting absorption after fat grafting has thus been demonstrated, no study has yet investigated the concentration of ADRCs to be incorporated. To enable the clinical use of ADRCs will require extracting cells in as simple a manner as possible and grafting of the appropriate concentration of cells. The Celution®800/CRS has been demonstrated to enable rapid grafting of the patient’s own freshly harvested ADRC. Lin et al. reported that cells from the Celution system are composed of heterogeneous cell populations including ADRCs (CD31- CD34+ CD45- CD90+ CD105- CD146-), endothelial (progenitor) cells (CD31+ CD34+ CD45- CD90+ CD105- CD146+), and vascular smooth muscle cells (CD31- CD34+ CD45- CD90+ CD105- CD146+) [5]. Accordingly, clinical trials of free fat grafting using the Celution system have begun in Europe [8]. Of the 67 patients treated, 50 reported satisfaction with treatment results after 12 months. There were no reported local cancer recurrences. Injection site cysts were reported as adverse events in ten patients [8]. The present study, in which the most appropriate concentration of cells extracted using the Celution®800/CRS was investigated, provides meaningful basic data for the future clinical use of ADRCs.

Lu et al. estimated that ADRC fat grafts transfected with vascular endothelial growth factor (VEGF) have the highest capillary density and graft survival [6]. This implies that reconstruction of the vasculature is important for the survival of fat grafts, and that VEGF may play a role in neovascularizing the recipient bed by paracrine signaling. Zhu et al. and Wang et al. also reported that normal ADRCs have high genetic expression and secrete large quantities of angiogenic factors, including VEGF [7,15]. Lu et al. also pointed out that enriching fat grafts with non-transfected ADRCs produced a significantly higher weight and capillary density of graft compared with the control groups that were treated with pure adipose tissue or without insulin [6]. Moseley et al. reported that the mass of fat with freshly isolated ADRCs was significantly greater than that of the adipose tissue alone group, with expression of endothelial marker (von Willebrand factor) and vascular support in the fat graft enriched with fresh ADRCs [14]. In the present study, it was also found that fat grafting with ADRCs significantly increased new blood vessels in grafted fat in all groups. We conjecture that the grafted ADRCs may have secreted VEGF and other angiogenic factors, resulting in vascularization.

In the present study, grafting of ≥10 times the amount of ADRCs in fat grafting caused tissue fibrosis and increased cyst formation in fat, with an increase in inflammatory cells, and fat absorption was also not inhibited. ADRCs have been reported to inhibit fibrosis of the liver [15], but there has been no previous report of fibrosis being caused by the grafting of large numbers of these cells. This study demonstrates that the concentration of transplanted ADRCs affects the engraftment and quality of the grafted fat.

We did not use < 3 × 10^5^ cells/mL of ADRCs in the current study. The results of the four groups in the present experiment indicated that 3 × 10^5^ cells/mL is the optimal concentration. Nonetheless, it is necessary to investigate the optimal number of cells by conducting additional experiments using ≤3 × 10^5^ cells/mL. The reason why grafting ≥10 times the amount of ADRCs in fat grafting led to tissue fibrosis and increased cyst formation in fat could be because the grafted human ADRCs had caused immunological reactions in BALB/C Jcl-nu/nu mice. Nude mice are hairless and are therefore convenient for human cell transplantation via injection. However, they lack only the T cells of the immune system. Previously, Shoshani et al. [11] and Lu et al. [6] used nude mice as a model for human ADRC transplantation and evaluated their fat absorption. Thus, this animal model has been established using this procedure. However, in light of the results from the present study, SCID mice that lack both T cells and B cells may be more appropriate for human ADRC transplantation than nude mice in the future.

In conclusion, the optimal ADRC concentration for free fat grafting enriched with human ADRCs extracted using the Celution®800/CRS was found to be 3 × 10^5^ cells per mL of fat, which effectively inhibits fat absorption. The results of this study may provide basic data for new free fat grafting methods in future plastic and reconstructive surgery.

## Competing interests

The authors declare they have not competing interests.

## Authors’ contributions

Conceived and designed the experiments: NK and NM. Performed the experiments: NK, YT, TO, SK, TH. Analyzed the data: NK and TO. Wrote the paper: NK and TO. Critical revision, organization and supervision: KK. All authors read and approved the final manuscript.
